# Case Report: Significant Clinical Benefit From Pemetrexed-Based Therapy in ROS1- and ALK-rearranged Lung Cancer With Adenosquamous Histology

**DOI:** 10.3389/fonc.2021.788245

**Published:** 2022-01-07

**Authors:** Tejas Patil, Yunan Nie, Dara L. Aisner, David Ross Camidge

**Affiliations:** ^1^ Division of Medical Oncology, Department of Medicine, University of Colorado, Anschutz Medical Campus, Aurora, CO, United States; ^2^ Department of Pathology, University of Colorado, Anschutz Medical Campus, Aurora, CO, United States

**Keywords:** ALK, ROS1, non-small-cell lung cancer, pemetrexed, squamous, adenosquamous

## Abstract

Pemetrexed (used as a platinum doublet or as a maintenance regimen) is an established therapy for patients with advanced non-squamous non-small-cell lung cancer (NSCLC). In addition, certain gene rearrangements (e.g., *ALK*, *ROS1*, *RET*) appear to especially benefit from the use of pemetrexed. Inferior outcomes with pemetrexed compared to other chemotherapies in patients with NSCLC demonstrating squamous histology removed these patients from the labeled indication for the drug. While most squamous cases do not harbor driver oncogenes, rare exceptions exist. Whether the poor outcomes with pemetrexed extend to NSCLC with squamous component harboring driver oncogenes remains unexplored. In this case series, we describe two patients with adenosquamous histology harboring an *ROS1* and *ALK* gene arrangement, respectively, who derived significant benefit from pemetrexed-based therapy. These cases suggest that the value of pemetrexed may need to be re-explored in adenosquamous NSCLC harboring such alterations.

## Introduction

First-line pemetrexed-platinum combinations and second-line pemetrexed monotherapy are licensed therapies for patients with metastatic non-squamous non-small-cell lung cancer (NSCLC) (1–4). The original monotherapy license granted in 2004 by the FDA was across all NSCLC histologies, but from 2008, differential efficacy data restricted its FDA-licensed indication across lines of therapy only to those NSCLC patients with non-squamous histology. In a phase III randomized study of first line cisplatin and pemetrexed versus cisplatin and gemcitabine in patients with locally advanced or metastatic NSCLC, the pemetrexed combination was associated with significantly better overall survival but only among those with adenocarcinoma or large cell histology (1). Among those with squamous histology, the gemcitabine doublet was associated with significantly better overall survival (1). Similar findings were seen in a phase III trial comparing pemetrexed with docetaxel in the second-line setting (2, 3).

Since the histology-based label restrictions on pemetrexed were implemented, molecular profiling of NSCLC for specific somatic driver oncogenes to predict benefit from targeted therapies has become commonplace, particularly in adenocarcinoma (4). Some of these oncogenes, most notably *ALK* and *ROS1* gene rearrangements, have also been associated with exaggerated sensitivity to pemetrexed (5, 6). While driver oncogenes are most commonly seen in conjunction with adenocarcinoma histology, they may also occur in cases of NSCLC with either squamous or adenosquamous histology, especially among patients with little or no smoking history (7, 8). Adenosquamous histology in NSCLC, representing a cancer displaying at least 10% of both adenocarcinoma and squamous cancer elements, is rare, representing <3% of all NSCLC cases (9). This mixed-histology subtype of NSCLC has the same reported EGFR and KRAS mutation rate as comparable adenocarcinoma cases, with the same driver mutations observed in both components (9). The allocation of adenosquamous cases was not explicitly mentioned in the pemetrexed treatment-by-histology analyses (3.4). While these cases may have been labeled as “other/unclassified” within the non-squamous group, it is also possible that cases with mixed histology were treated as either categorically as squamous or non-squamous based on the predominant histology seen (3, 4).

NCCN and IASLC/AMP/CAP testing guidelines have now all included wording that would support molecular testing in adenosquamous and consideration of molecular testing in all squamous cases (10, 11). Whether patients with a driver oncogene associated with exaggerated sensitivity to pemetrexed respond as such when their cancer is associated with squamous or adenosquamous histology remains largely unknown. Here, we describe two patients with adenosquamous NSCLC histology harboring an *ROS1* and *ALK* gene arrangement, respectively, who derived significant clinical benefit from treatment with pemetrexed.

## Case Description

### Case 1

A 58-year-old male former smoker (estimated 12 pack years, quit 20 years prior to diagnosis) presented to his primary care doctor for malaise, fever, and a cough with a 20-pound weight loss. He was initially diagnosed with pneumonia and treated with antibiotics. When his symptoms did not improve, he presented to the hospital for further evaluation. On admission, he was hypoxemic (SpO2 85%) requiring supplemental oxygen. His physical exam was notable for sinus tachycardia and dullness to percussion along right lower lung fields. Subsequent imaging to rule out pulmonary embolism using a computed tomography (CT) scan failed to identify a pulmonary embolus but did find a right lower lobe mass, small right pleural effusion, and right hilar and mediastinal lymphadenopathy. A subsequent PET-CT showed intense FDG-avidity within the right lower lobe mass, mediastinal nodes, and within the right pleural effusion. A brain MRI was negative for intracranial metastases. He underwent excisional biopsy of an upper paratracheal lymph node and was subsequently diagnosed with NSCLC. Immunohistochemical (IHC) staining was positive for CK7 and TTF-1, focally positive for p63 and CK20, and negative for CDX2, supportive of an adenosquamous subtype. He underwent molecular testing through next-generation sequencing (NGS) and was found to have a *CD74-ROS1* fusion.

**Figure 1:**
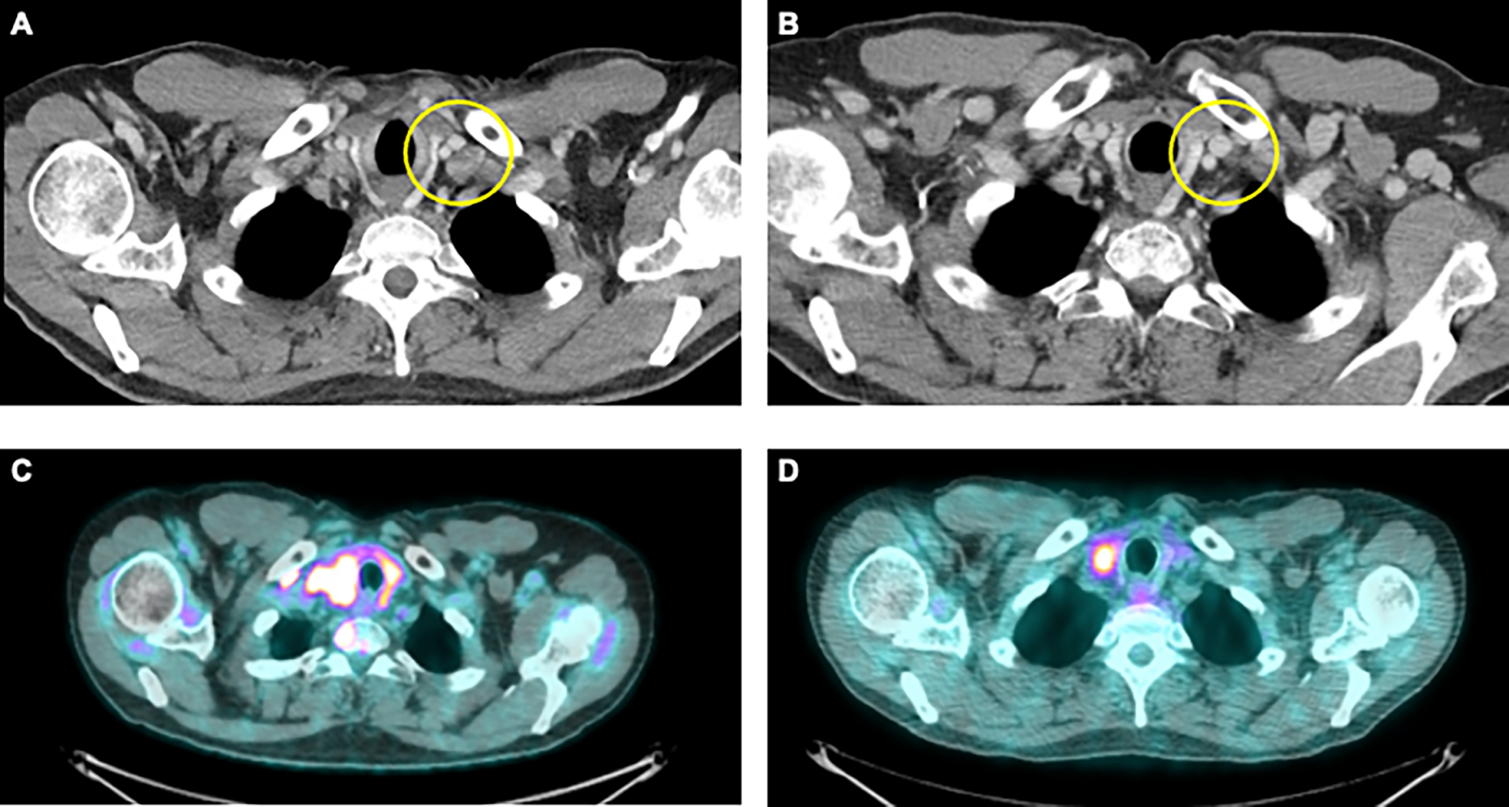
Representative images from patients in this case series. **(A)** CT imaging from a 58-year-old male former smoker with a diagnosis of stage IVB NSCLC with an adenosquamous histology harboring a *CD74-ROS1* fusion upon progression on entrectinib and prior to use of carboplatin, pemetrexed and pembrolizumab. **(B)** CT imaging of best-treatment response with significant reduction in the size of the left supraclavicular lymph node. **(C)** PET/CT imaging from a 40-year-old male never smoker with stage IVB NSCLC with an adenosquamous histology harboring an *EML4-ALK* fusion prior to treatment with single agent pemetrexed. **(D)** PET/CT imaging demonstrating significant improvement within known right paratracheal nodes.

The patient was initially started on entrectinib 600 mg daily. He did well with this therapy but had mediastinal lymph node progression after 8 months of starting treatment. He switched to second line therapy using carboplatin, pemetrexed, and pembrolizumab. His first on-treatment scan demonstrated a partial response (**Figure 1**) . He completed five cycles of carboplatin, pemetrexed, pembrolizumab and was then transitioned to maintenance therapy with pemetrexed and pembrolizumab. He received 16 cycles of pemetrexed and pembrolizumab maintenance therapy prior to progression of disease.

### Case 2

A 40-year-old male never smoker presented to the hospital after developing acute chest pain on a commercial flight. Initial investigations using a CT pulmonary embolus protocol in the hospital identified a right-sided pulmonary embolism, but also revealed a left superior sulcus mass with left hilar and mediastinal lymphadenopathy. A brain MRI did not show any intracranial metastases at that time. He underwent a CT-guided biopsy of the left upper lobe mass that demonstrated poorly differentiated carcinoma, morphologically favoring squamous NSCLC. There was insufficient tissue for full immunohistochemical or molecular analyses. A subsequent PET/CT revealed no new sites of disease, and he was initially staged as having cT2N2M0, stage IIIA superior sulcus NSCLC. He received neoadjuvant chemoradiation with cisplatin and etoposide and 5,000 cGy over 25 fractions using the SWOG 8805 regimen. This was followed by a left upper lobectomy and mediastinal lymph node sampling. Surgical pathology identified a 4.9 × 3.7cm tumor described diagnosed as squamous cell carcinoma based on morphologic features (no IHC performed) with lymphovascular invasion. Carcinoma was present in level 5 and 6 lymph nodes, consistent with pathologic N2 disease.

He received four cycles of adjuvant docetaxel. Approximately 4 months after completion of adjuvant therapy, the patient began experiencing headaches and MRI of the brain demonstrated seven bilateral cerebral and left cerebellar brain lesions. He received whole brain radiation therapy (WBRT) to 3,000 cGy followed by stereotactic radiosurgery (SRS) at 2,500 cGy to the brain lesions. Given his lifelong history of no tobacco use, his pulmonary resection sample was tested, and he was found to be ALK positive for ALK expression (using the D5F3 antibody), and subsequently *EML4-ALK* fusion was confirmed by NGS. Owing to lack of measurable disease, he did not receive crizotinib in the context of a clinical trial, as this drug was not yet approved. In the setting of minimal active extracranial disease, he received approximately 6 months of vinorelbine and bevacizumab, followed by 6 months of bevacizumab maintenance therapy at an outside institution. He subsequently developed progressive disease within his left lung.

At that point he switched to single-agent pemetrexed, despite the putative squamous histology, with a minor response in his lung nodules. Importantly, he remained on this therapy for a total of 12 months. He discontinued pemetrexed once crizotinib was FDA approved and commercially available, but this switch was not due to overt radiographic progression. He developed CNS progression on crizotinib and was switched to brigatinib (in the context of a clinical trial). Four years after switching to brigatinib, he developed symptomatic occipital brain metastases that prompted neurosurgical resection. Surgical pathology revealed poorly differentiated carcinoma that was positive for p63, Napsin A, and TTF1 and negative for CK5/CK6—findings suspicious for adenosquamous histology. Next-generation sequencing confirmed an *EML4-ALK* gene rearrangement, consistent with prior molecular testing.

## Discussion

At this time, pemetrexed therapy is not licensed for use in patients with NSCLC and squamous histology (1–3). Its use among those with adenosquamous histology is less well-defined with individual practitioners conceivably making choices based on the dominant histology in the patient’s sample on a case-by-case basis. However, as molecular subtypes of NSCLC associated with exaggerated sensitivity to pemetrexed, notably *ALK* and *ROS1* gene-rearranged disease, are known to co-exist across histologies including squamous and adenosquamous, albeit at differing frequencies, it becomes important to ask whether the histology or the driver oncogene will dominate the cancer’s sensitivity to pemetrexed (5–9). The two patients in our series harbored an *ROS1* and *ALK* gene rearrangement in the setting of NSCLC labeled as either squamous (based on morphology alone) or adenosquamous histology (based on IHC testing). Both demonstrated prolonged clinical benefit and prolonged responses from pemetrexed-containing regimens, far exceeding the median PFS reported in past studies evaluating pemetrexed in patients with squamous histology (1–3). While the first case did receive pemetrexed together with carboplatin and pembrolizumab, continuing with continuation of the pembrolizumab with the pemetrexed during the prolonged use of maintenance therapy, gene-rearranged NSCLC is often considered hyporesponsive to immunotherapy (12). This suggests that the patient’s prolonged response was likely at least in part due to pemetrexed rather than solely immunotherapy. The second patient did not receive pembrolizumab with pemetrexed, again suggesting that the response was due to chemotherapy effects.

Adenosquamous carcinoma of the lung is defined as having at least 10% of both histologic types, in the absence of other separately defined histologic subtypes. Most cases utilize immunohistochemistry to help make their pathological diagnoses (13). However, histological categorization of NSCLC remains liable to subjective aspects of interpretation. The diagnosis of adenosquamous can rely on the identification of a minor and sometimes subtle subset of tumor cells, which may only be fully recognized with immunohistochemistry. Particularly when considered in conjunction with the potential for adenocarcinoma to demonstrate positive staining for p63, distinguishing adenocarcinoma, squamous cell carcinoma, and adenosquamous carcinoma can be subject to different interpretations (14). More recently, antibodies such as p40 have been found to be more specific for squamous cell carcinoma, thus decreasing diagnostic uncertainty (15). Whether the histological diagnoses in these cases would stand up to retrospective evaluation considering the molecular findings, clinical history, and availability of new immunohistochemical stains is uncertain. Equally, as the most definitive diagnosis of “squamous” cancer in the second case was derived from resected, previously irradiated CNS tissue, the impact of the prior radiation therapy on the microscopic appearance and immunohistochemical profile of the cancer also must be considered.

Nevertheless, on a pragmatic level, if such cases are being called squamous or adenosquamous in the real world and decisions about the applicability or exclusion of pemetrexed-based therapy are being made on such designations, our cases illustrate the need to re-explore the value of histology-based pemetrexed decisions in the setting of a known driver oncogene. In one series, the frequency of EGFR mutations detected within cases of adenosquamous carcinoma in an East Asian population was 55%, with high convergence mutation rate in both adenomatous and squamous components (16). We are aware of one previous case report describing successful pemetrexed therapy in a patient with NSCLC harboring an EGFR mutation in the setting of adenosquamous histology (17). Together with our series commenting on pemetrexed sensitivity in patients with purported squamous or adenosquamous NSCLC and an underlying *ALK* and *ROS1* gene rearrangement, we would suggest that, while further study is required, at present the information derived from the driver oncogene should not be automatically deprioritized versus the information derived from the cancer’s histological appearance when deciding for or against the use of pemetrexed as a therapy.

## Data Availability Statement

The original contributions presented in the study are included in the article/supplementary material. Further inquiries can be directed to the corresponding author.

## Ethics Statement

Ethical review and approval was not required for the study on human participants in accordance with the local legislation and institutional requirements. The patients/participants provided their written informed consent to participate in this study. Written informed consent was obtained from the individual(s) for the publication of any potentially identifiable images or data included in this article.

## Author Contributions

YN: Design of the work, acquisition of data, analysis of data, interpretation of data, drafting the work, revising the work critically for important intellectual content, final approval of the version to be published, agreeing to be accountable for all aspects of the work in ensuring that questions related to the accuracy or integrity of any part of the work are appropriately investigated and resolved. DA: Revising the work critically for important intellectual content, final approval of the version to be published, agreeing to be accountable for all aspects of the work in ensuring that questions related to the accuracy or integrity of any part of the work are appropriately investigated and resolved. DC: Conception of the work, design of the work, interpretation of data, drafting the work, revising the work critically for important intellectual content, final approval of the version to be published, agreeing to be accountable for all aspects of the work in ensuring that questions related to the accuracy or integrity of any part of the work are appropriately investigated and resolved. TP: Conception of the work, design of the work, acquisition of data, analysis of data, interpretation of data, drafting the work, revising the work critically for important intellectual content, final approval of the version to be published, agreeing to be accountable for all aspects of the work in ensuring that questions related to the accuracy or integrity of any part of the work are appropriately investigated and resolved.

## Funding

This project was supported in part by the University of Colorado Lung Cancer Specialized Program of Research Excellence (P50CA058187) and the University of Colorado Cancer Center Thoracic Oncology Research Initiative. Data collection was made possible in part by the NIH/NCATS Colorado CTSA Grant (UL1 TR002535).

## Conflict of Interest

The authors declare that the research was conducted in the absence of any commercial or financial relationships that could be construed as a potential conflict of interest.

## Publisher’s Note

All claims expressed in this article are solely those of the authors and do not necessarily represent those of their affiliated organizations, or those of the publisher, the editors and the reviewers. Any product that may be evaluated in this article, or claim that may be made by its manufacturer, is not guaranteed or endorsed by the publisher.

## References

[1] Scagliotti GV Parikh P von Pawel J Biesma B Vansteenkiste J Manegold C. Phase III Study Comparing Cisplatin Plus Gemcitabine With Cisplatin Plus Pemetrexed in Chemotherapy-Naive Patients With Advanced-Stage non-Small-Cell Lung Cancer. J Clin Oncol (2008) 26(21):3543–51. doi: 10.1200/JCO.2007.15.0375 18506025

[2] Scagliotti G Hanna N Fossella F Sugarman K Blatter J Peterson P. The Differential Efficacy of Pemetrexed According to NSCLC Histology: A Review of Two Phase III Studies. Oncologist (2009) 14(3):253–63. doi: 10.1634/theoncologist.2008-0232 19221167

[3] Scagliotti G Brodowicz T Shepherd FA Zielinski C Vansteenkiste J Manegold C. Treatment-By-Histology Interaction Analyses in Three Phase III Trials Show Superiority of Pemetrexed in Nonsquamous non-Small Cell Lung Cancer. J Thorac Oncol (2011) 1:64–70. doi: 10.1097/JTO.0b013e3181f7c6d4 21119545

[4] Waterhouse DM Tseng WY Espirito JL Robert NJ. Understanding Contemporary Molecular Biomarker Testing Rates and Trends for Metastatic NSCLC Among Community Oncologists. Clin Lung Cancer (2021) 21):S1525–7304. doi: 10.1016/j.cllc.2021.05.006 34187757

[5] Camidge DR Kono SA Lu X Okuyama S Baròn AE Oton AB. Anaplastic Lymphoma Kinase Gene Rearrangements in Nonsmall Cell Lung Cancer are Associated With Prolonged Progression-Free Survival on Pemetrexed. J Thorac Oncol (2011) 6(4):774–80. doi: 10.1097/JTO.0b013e31820cf053 PMC3626562 21336183

[6] Chen YF Hsieh MS Wu SG Chang YL Yu CJ Yang JC. Efficacy of Pemetrexed-Based Chemotherapy in Patients With ROS1 Fusion-Positive Lung Adenocarcinoma Compared With in Patients Harboring Other Driver Mutations in East Asian Populations. J Thorac Oncol (2016) 11(7):1140–52. doi: 10.1016/j.jtho.2016.03.022 27094798

[7] Caliò A Nottegar A Gilioli E Bria E Pilotto S Peretti U. ALK/EML4 Fusion Gene may be Found in Pure Squamous Carcinoma of the Lung. J Thorac Oncol (2014) 9(5):729–32. doi: 10.1097/JTO.0000000000000109 24722159

[8] Watanabe J Togo S Sumiyoshi I Yukiko N Suina K Mizuno T. Clinical Features of Squamous Cell Lung Cancer With Anaplastic Lymphoma Kinase (ALK)-Rearrangement: A Retrospective Analysis and Review. Oncotarget (2018) 9(35):24000–13. doi: 10.18632/oncotarget.25257 PMC5963613 29844868

[9] Borczuk AC. Uncommon Types of Lung Carcinoma With Mixed Histology: Sarcomatoid Carcinoma, Adenosquamous Carcinoma, and Mucoepidermoid Carcinoma. Arch Pathol Lab Med (2018) 142(8):914–21. doi: 10.5858/arpa.2017-0584-RA 30040455

[10] National Comprehensive Cancer Network (NCCN). NCCN Clinical Practice Guidelines in Oncology: Non-Small Cell Lung Cancer. Version 1.2022. Accessed November 10, 2021. 10.6004/jnccn.2021.0058 PMC10203822 34902832

[11] Kalemkerian GP Narula N Kennedy EB Biermann WA Donington J Leighl NB. Molecular Testing Guideline for the Selection of Patients With Lung Cancer for Treatment With Targeted Tyrosine Kinase Inhibitors: American Society of Clinical Oncology Endorsement of the College of American Pathologists/International Association for the Study of Lung Cancer/Association for Molecular Pathology Clinical Practice Guideline Update. J Clin Oncol (2018) 36(9):911–9. doi: 10.1200/JCO.2017.76.7293 29401004

[12] Camidge DR Doebele RC Kerr KM. Comparing and Contrasting Predictive Biomarkers for Immunotherapy and Targeted Therapy of NSCLC. Nat Rev Clin Oncol (2019) 16(6):341–55. doi: 10.1038/s41571-019-0173-9 30718843

[13] Terry J Leung S Laskin J Leslie KO Gown AM Ionescu DN. Optimal Immunohistochemical Markers for Distinguishing Lung Adenocarcinomas From Squamous Cell Carcinomas in Small Tumor Samples. Am J Surg Pathol (2010) 34(12):1805–11. doi: 10.1097/PAS.0b013e3181f7dae3 21107086

[14] Rekhtman N Ang DC Sima CS Travis WD Moreira AL. Immunohistochemical Algorithm for Differentiation of Lung Adenocarcinoma and Squamous Cell Carcinoma Based on Large Series of Whole Tissue Sections With Validation in Small Specimens. Mod Pathol (2011) 24(10):1348–59. doi: 10.1038/modpathol.2011.92 21623384

[15] Bishop JA Teruya-Feldstein J Westra WH Pelosi G Travis WD Rekhtman N. P40 (Δnp63) is Superior to P63 for the Diagnosis of Pulmonary Squamous Cell Carcinoma. Mod Pathol (2012) 25(3):405–15. doi: 10.1038/modpathol.2011.173 22056955

[16] Shi X Wu H Lu J Duan H Liu X Liang Z. Screening for Major Driver Oncogene Alterations in Adenosquamous Lung Carcinoma Using PCR Coupled With Next-Generation and Sanger Sequencing Methods. Sci Rep (2016) 6:22297. doi: 10.1038/srep22297 26923333 PMC4770439

[17] Watanabe H Tamura T Kagohashi K Kawaguchi M Kurishima K Satoh H. Successful Pemetrexed-Containing Chemotherapy for Epidermal Growth Factor Receptor Mutation-Positive Adenosquamous Cell Carcinoma of the Lung: A Case Report. Mol Clin Oncol (2016) 4(4):628–30. doi: 10.3892/mco.2016.756 PMC4812476 27073680

